# Palpable Mass on the Head after Minor Trauma

**DOI:** 10.1155/2016/1340589

**Published:** 2016-11-10

**Authors:** K. M. Nikolakopoulos, C. P. Papageorgopoulou, I. G. Ntouvas, S. Kakkos, I. Tsolakis

**Affiliations:** Department of Vascular Surgery, University of Patras, Patras, Greece

## Abstract

Temporal artery is superficially exhibited and easily traumatized. Rarely, a minor and blunt trauma, especially in elderly who are under anticoagulants, can cause a pseudoaneurysm. Diagnosis should be based, primarily, on history and physical examination and secondarily on duplex ultrasound scanning which will lead to confirmation and preoperative planning. The therapeutical plan consists of surgical ligation and excision of the aneurysm. Surgery can be performed under local anesthesia with no postoperative major or minor complications. Endovascular approach consists of catheter embolization and remains a second option due to the risk of complications and the inconclusive results. On this review, authors present a case of an 80-year-old male with a pseudoaneurysm of superficial temporal artery.

## 1. Introduction

Temporal artery is superficially exhibited and easily traumatized. Rarely, a minor and blunt trauma, especially in elderly who are under anticoagulants, can cause a pseudoaneurysm. The diagnosis is based on history and physical examination. However, duplex ultrasound scanning [1], contrast computed tomography (CTA), and angiography can help in the confirmation and preoperative planning. This report presents a patient with a pseudoaneurysm secondary to a minor, blunt trauma.

## 2. Case Presentation

**Figure 1:**
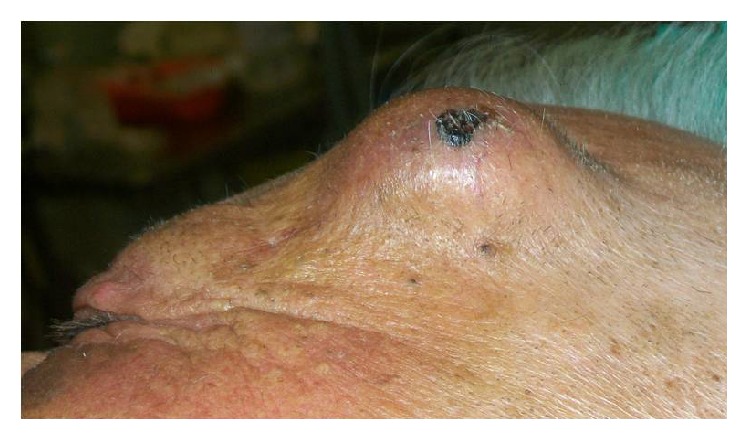
Pulsatile mass at the temporal side of the left frontal lobe.

**Figure 2:**
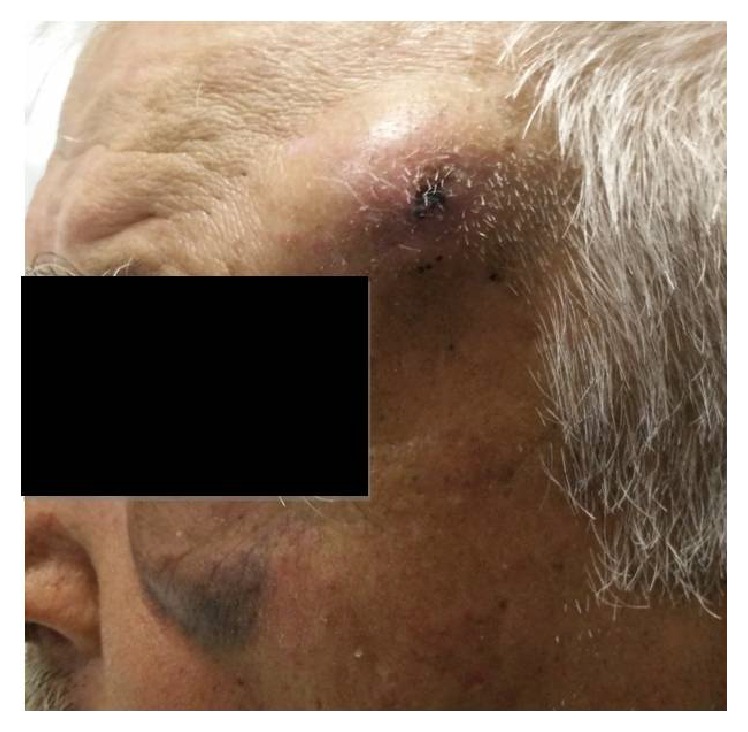
Pseudoaneurysm with hematoma extended into the periorbital space.

An 80-year-old male presented to the emergency department of our hospital with a mass on the left frontal bone, after a minor fall on the ground ten days ago. The patient was under anticoagulants for his chronic atrial fibrillation at therapeutic levels. Examination revealed pale skin with necrotic components overlying a 1.56 × 1.12 cm pulsatile mass on the temporal side of the left frontal bone (Figures 1 and 2).

**Figure 3:**
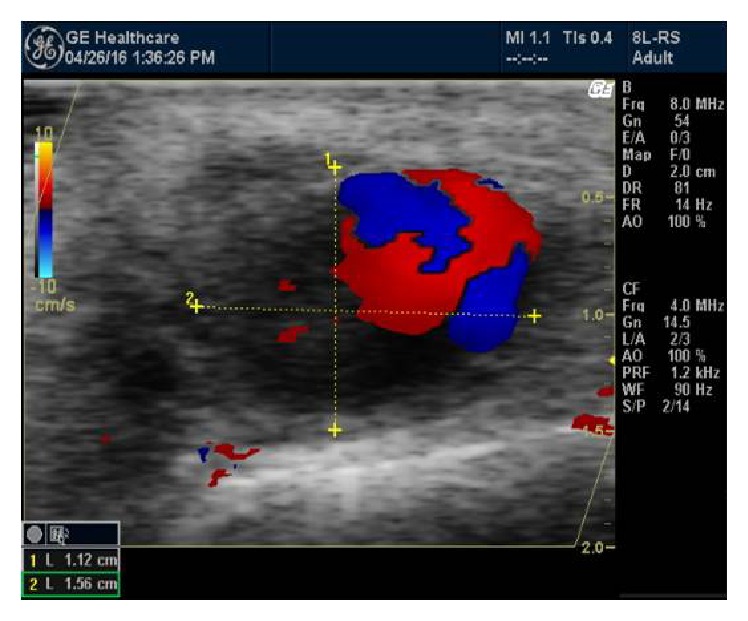
Ying and Yang sing.

**Figure 4:**
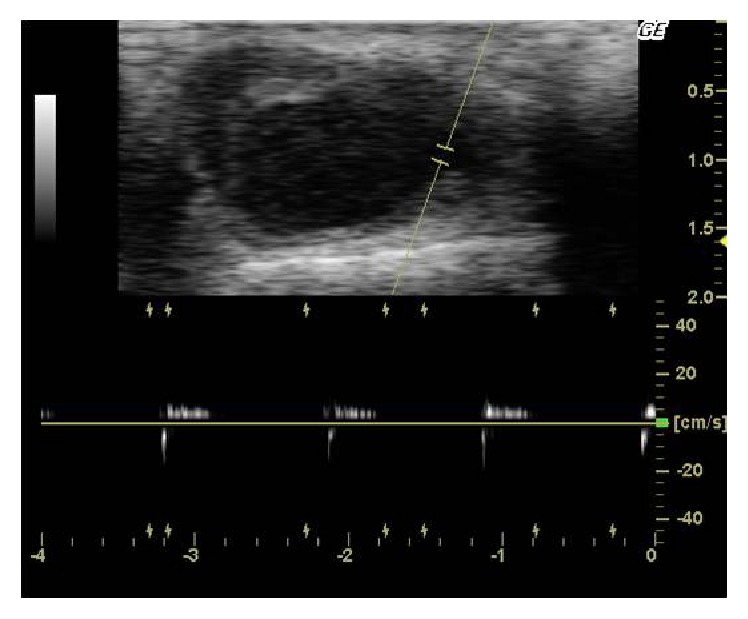
“To and fro” pattern.

A duplex scanning was performed confirming the initial diagnosis of the pseudoaneurysm of the superficial temporal artery (Figures 3 and 4).

Differential diagnosis includes the following: pseudoaneurysm, abscess, cyst, lipoma, simple hematoma, vascular tumor, and true aneurysm [2, 3]. Based on the findings of the ultrasound the patient's diagnosis was pseudoaneurysm of the superficial temporal artery.

**Figure 5:**
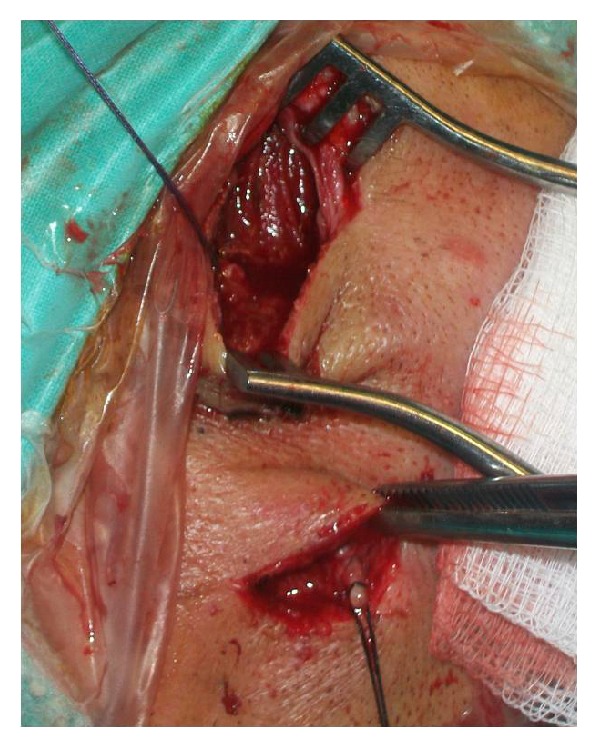
Proximal and distal control of the temporal artery and excision of the aneurysm.

The patient was operated on under local anesthesia at the same day. Arterial control was achieved by exposing the proximal and distal segments of the artery. Ligation and excision of the aneurismal artery were performed (Figure 5). The necrotic tissue was removed and primary closure of the wound was performed. The patient recovered well and was discharge the same day. No recurrence or complications were recognized at one and three months after the procedure.

## 3. Discussion

Pseudoaneurysm is defined as a dilatation of an artery that includes a defect in one or more (less than three) layers of the arterial wall. Pseudoaneurysm of the superficial temporal artery, caused by minor blunt trauma, was first described by Bartholin in 1740 and ever since represents the 1% of all traumatic aneurysms [1, 4]. Due to its course, superficially and beyond the frontal bone, blunt trauma to the temple can cause a defect at the artery as it is compressed against the bone, especially in elderly patients who may have atherosclerotic and/or calcified arteries or who are on anticoagulants or antiplatelet agents. Men are more vulnerable to that type of aneurysm, because of their increased incidence of trauma [5].

The diagnosis is based on history taking and physical examination. The typical case starts with a minor blunt trauma to the temple one to six weeks ago [6, 7]. The typical patient presents with a tender, pulsatile, compressible mass on the temporal or frontal bone with or without patches of necrosis. Bruits may be auscultated. The mass is easily compressible and no pulses are recognized when the temporal artery is compressed proximally. There is a possibility of hematoma up to the periorbital or subgaleal space.

Differential diagnosis includes abscess, cyst, lipoma, simple hematoma, vascular tumor, and true aneurysm [2, 3]. In order to confirm or exclude the diagnosis, a duplex ultrasound scanning should be performed. Ultrasound provides an excellent resolution on superficial vessels and easily defines the presence of pseudoaneurysm (yin-yang sign, “to and fro” pattern). Historically, angiography was used, with major complications such as stroke and vascular injury. Many authors have used computed tomography (CTA) for the diagnosis.

The standard therapeutic plan is surgery. However, “watch and wait” treatment was suggested, but the long duration of symptoms, headaches, and risk of rupture discouraged its use [8]. Surgical treatment consists of proximal and distal ligation of the temporal artery and excision of the aneurysmatic artery and sac [1]. Primary repair of the artery has been described in small puncture or tear on a healthy vessel [9]. Due to the rich collateral blood supply of the face and skull, the ligation remains the first surgical option. More recently, nonsurgical treatments have been used such as conservative treatment, thrombin injection, and endovascular embolization or coiling. Every method has its advantages and disadvantages and should be chosen based on the size of the pseudoaneurysm, chronicity, patient's clinical status, and preference. Conservative treatment which is simply compression of the pseudoaneurysm will lead to thrombus creation inside the lumen, reducing the size of the aneurysm. Continuous compression [10] should be attempted on pseudoaneurysms without active bleeding and small size. However, there are the risks of rupture, bleeding, and thromboembolism. Percutaneous ultrasound-guided injection of thrombin, a basic component of the pathway of coagulation, can be used on chronic or subacute pseudoaneurysms of size less than 4 cm in diameter [7]. This technique is important for patients who want to minimize the risk of the surgical scar. Two to three months are needed for the resolution of the thrombosed pseudoaneurysm and reexamination with duplex ultrasound should be followed, in order to ensure that there is no recanalization of the pseudoaneurysm. Complications of the procedure include embolization of nontarget vessels leading to ischemic stroke, seizures, and occlusion of feeding vessels. Endovascular coiling, embolization, or stenting is recommended in complicated cases of acute trauma or in deep branches, difficult to approach, on hemodynamically stable patient [10]. When angiography is used for the diagnosis, endovascular approach should be performed in first place.

We conclude the following:

1. The case presented illustrates and binds the preoperative ultrasound image of the pseudoaneurysm with the real, operative one.
2. Temporal artery pseudoaneurysm is a rare clinical presentation. Diagnosis should be based, primarily, on history and physical examination and secondarily on duplex ultrasound scanning which will lead to confirmation and preoperative planning.
3. The therapeutical plan consists of surgical ligation and excision of the aneurysm.
